# Relationships between Serum Biomarkers of Oxidative Stress and Tobacco Smoke Exposure in Patients with Mental Disorders

**DOI:** 10.3390/antiox12061299

**Published:** 2023-06-19

**Authors:** Ana-Maria Vlasceanu, Daniela Gradinaru, Miriana Stan, Viorela G. Nitescu, Daniela Luiza Baconi

**Affiliations:** 1 Department of Toxicology, Faculty of Pharmacy, Carol Davila University of Medicine and Pharmacy, 37 Dionisie Lupu Street, Sector 2, 20021 Bucharest, Romania; ana.vlasceanu@umfcd.ro (A.-M.V.); miriana.stan@umfcd.ro (M.S.); daniela.baconi@umfcd.ro (D.L.B.); 2 Department of Biochemistry, Faculty of Pharmacy, Carol Davila University of Medicine and Pharmacy, 37 Dionisie Lupu Street, Sector 2, 20021 Bucharest, Romania; 3 Emergency Clinical Hospital for Children Grigore Alexandrescu, Pediatric Clinic 2, Ward ATI -Toxicology, 30-32 Iancu de Hunedoara Street, 20021 Bucharest, Romania; viorela.nitescu@umfcd.ro

**Keywords:** cotinine, nicotine, oxidative stress, glutathione, cigarette smoking, antioxidants

## Abstract

The role of cigarette smoking as an aggravating factor of systemic oxidative stress in patients with mental disorders has not been extensively investigated, although significantly higher rates of smoking are recorded in these subjects in comparison with the general population. In the present study, we tested the hypothesis that smoking might be an exacerbator of systemic oxidative stress, being directly correlated with the degree of exposure to tobacco smoke. We analyzed, in 76 adult subjects from a public health care unit, the relationships between serum cotinine levels as a marker of tobacco smoke exposure, and three biomarkers of oxidative stress: the serum glutathione (GSH), the advanced oxidation protein products (AOPPs), and the total serum antioxidant status (FRAP). The results indicate that the degree of tobacco smoke exposure was inversely associated with GSH levels in both passive and active smokers, suggesting that smoke particulate components’ toxicity is associated with a systemic GSH depletion. Paradoxically, the lowest AOPP levels which were positively associated with GSH, were recorded in active smoking patients whereas in passive smokers individual values of AOPPs decreased along with the increase in GSH levels. Our data suggest that an enhanced inhalation of particulate constituents of cigarette smoke could induce critical changes in systemic redox homeostasis and GSH can no longer exert its antioxidant role.

## 1. Introduction

Long-term exposure to constituents in tobacco smoke causes oxidative stress and subsequently induces cell damage through lipid peroxidation, depletion of plasma antioxidants (such as vitamin A and C), and inflammatory response, reflected by increased levels of C-reactive protein, fibrinogen, and interleukin-6 (IL-6) [1,2]. The systemic oxidative stress observed in smokers can occur directly, as a result of exposure to oxidants—mainly polycyclic aromatic hydrocarbons (PAHs) and volatile organic compounds (VOCs)—or indirectly, through inflammation mechanisms [3,4,5]. The indirect oxidative stress is predominantly caused by the action of macrophages and dendritic cells attempting to absorb and dissolve or detoxify the inhaled particulate matter [6]. The release of reactive oxygen species (ROS) at the site of damage and inflammation helps to combat foreign pathogens and restores the injured tissue while simultaneously increasing the burden of oxidative stress [7]. Nicotine is an addictive substance that is a constituent of inhaled cigarette smoke [8]. Cotinine is a metabolite of nicotine exposure that is often used as a biomarker of nicotine-containing smoke exposure [9]. Recently, it was shown that high cotinine levels were associated with an increase in the inflammatory process and oxidative stress in the vasculature of smokers, which is characterized by high IL-6 expression and low superoxide dismutase (SOD) expression [10]. It is known that in subjects with mental disorders such as ADHD, anxiety disorders, and depression, significantly higher rates of smoking are recorded in comparison to the general population, suggesting a strong relationship between nicotine addiction and symptomatology of these disorders [11]. Furthermore, numerous studies have indicated that smoking behavior and nicotine dependence could increase the risk of developing major depressive disorder [12]. Patients with mental disorders use cigarette smoking to deal with their emotions and mood swings [13]. New data highlight the negative consequences of smoking or nicotine addiction specifically in subjects with mental disorders, beyond the inflammation and oxidative stress which could be a common characteristic for all smokers [14,15]. This relationship is very complex, as smokers can also develop a broad spectrum of non-communicable diseases [16]. Although this risk is influenced by other individual determinants such as the genome, epigenome, exposome, microbiome, and others, smokers with serious mental illness have an increased risk of dying from cancer, lung disease, and cardiovascular disease, and account for more than 200,000 out of the 520,000 tobacco-related deaths each year [16,17,18]. Individuals with serious mental diseases die about 15 years earlier than individuals without serious mental diseases who never smoke [19]. About half of the deaths among those hospitalized for schizophrenia, depression, or bipolar disorder are from causes linked to smoking [18].

Therefore, in the present study we tested the hypothesis that, in subjects with mental disorders, smoking might be an exacerbator of systemic oxidative stress, which is directly correlated with the degree of exposure to tobacco smoke. For this purpose, the relationships between serum cotinine levels as a marker of tobacco smoke exposure, and three biomarkers relevant for systemic oxidative stress evaluation, namely: (1) the serum glutathione (GSH), as a redox status biomarker; (2) the advanced oxidation protein products (AOPPs), as markers of serum protein oxidative damage; and (3) the total serum antioxidant status, measured as ferric reducing antioxidant power (FRAP) were analyzed in a group of adult subjects from a public mental health care unit.

## 2. Materials and Methods

### 2.1. Participants and Study Design

The cross-sectional research study was conducted on 76 patients (61 men and 15 women), with an age range of 25–40 years, mean age 50 ± 20.48 years, selected from the patients hospitalized in the Sf. Maria Psychiatry Hospital, at Vedea, the Argeş Department, Romania. The research study was approved by the Ethics Committee of the Sf. Maria Psychiatry Hospital, and written consent was obtained from every patient. The presence of a mental disorder, a healthy status, and no antioxidant supplements in the treatment regimen were set as inclusion criteria. The subjects—smokers and non-smoker individuals—were diagnosed with schizophrenia, bipolar disorder, or major depressive disorder. The exclusion criteria were comorbidities such as hypertension, diabetes mellitus or dyslipidemia, obesity, endocrine diseases, severe hematological, renal, or hepatic diseases. In addition, the absence of intellectual disability by history, and the absence of HIV-1 infection or a serious medical disorder that would affect cognitive functioning were taken into consideration.

The degree of exposure to active or passive tobacco smoke was detected by measuring the level of serum cotinine—a metabolite of nicotine—which indicates the level of nicotine exposure in the human body for 3–4 days [20,21].

A single blood sample (6 mL) was obtained from each subject after 8–12 h of overnight fasting. The serum samples were separated by centrifugation (3000× *g* × 10 min) from whole blood for the assessment of biochemical parameters.

### 2.2. Biochemical Parameters

Cotinine levels were detected using an enzyme-linked immunosorbent assay (ELISA) kit (LifeSpan BioSciences, Lynnwood, WA, USA, catalog no. LS-F25700) based on a competitive immunoenzymatic reaction in which cotinine present in the serum sample competes with the pre-coated cotinine in the well micro-plate for the binding sites of the biotin-conjugated detection antibody. The tests were performed on a ChemWell 2190 Analyzer (Awareness Technology, Palm City, FL, USA). The inter-assay coefficient of variation (CV) was 5.3% and the intra-assay CV was 2.7%.

The assay of GSH, with the reagent 5,5′-ditiobis-2-nitrobenzoic acid (DTNB), was performed by following a standard Ellman’s method. Briefly, 2.3 mL of potassium phosphate (0.2 M, pH 7.6) buffer was taken in the cuvette, followed by the addition of 0.2 mL of serum. To the serum, 0.5 mL of DTNB (0.001 M) in a buffer was added. The absorbance of the reaction product in the cuvette was read after 5 min at 412 nm using a Shimadzu 1601 UV/Visible double beam spectrophotometer, and the GSH level was determined from a standard curve with concentrations between 5 and 100 μM GSH [22].

The serum advanced oxidation protein products (AOPPs) were measured spectrophotometrically at 340 nm using a commercially available assay kit (OxySelect STA-318, Cell Biolabs, San Diego, CA, USA). The concentration of AOPPs was expressed in chloramine T equivalents (µmol/L). The tests were performed on a ChemWell 2190 Analyzer (Awareness Technology, Palm City, FL, USA). The inter-assay and intra-assay coefficients of variation were 5.7% and 8.4%, respectively.

The measurement of the ferric reducing antioxidant power (FRAP) was conducted using the method described by Jansen and Ruskovska, adapted and optimized from the original method described by Benzie and Strain [23,24]. The reduction of the ferric-tripyridyltriazine (FeIII-TPZ) complex to the ferrous form (FeII-TPZ) in the presence of antioxidants develops an intense blue color, whose absorbance was measured at 600 nm. The tests were performed on a ChemWell 2190 Analyzer (Awareness Technology, Palm City, FL, USA). The inter-assay coefficient of variation (CV) was 4.9% and the intra-assay CV was 5.2%.

### 2.3. Statistical Analysis

We used the Statistical Package for Social Sciences software (SPSS, version 15, Chicago, IL, USA). The results for serum cotinine and oxidative stress biomarkers (GSH, FRAP, and AOPPs) are presented as mean + standard deviation. Comparison between tertile subgroups was performed using Student’s unpaired t-test. Pearson’s two-tailed bivariate correlation (r) or Spearman’s rank order (rho) correlation analyses were performed to examine associations of the individual levels of serum cotinine with the oxidative stress biomarkers GSH, FRAP, and AOPP. The level of significance was set at 0.05.

## 3. Results

The characterization of the study group, including socio-demographic and clinical characteristics, is presented in Table 1. Out of the total of 76 patients included in the study, the majority were men (61 patients, which represents 80.26%) and a small number were women (15 patients, representing 19.74%), with the ratio of men to women being 4.06. Paranoid schizophrenia was the most frequent diagnosis (46.05% of patients). Antipsychotics and anxiolytics were in the treatment regimen, as well as carbamazepine, which was administered to all of the patients.

**Figure 1:**
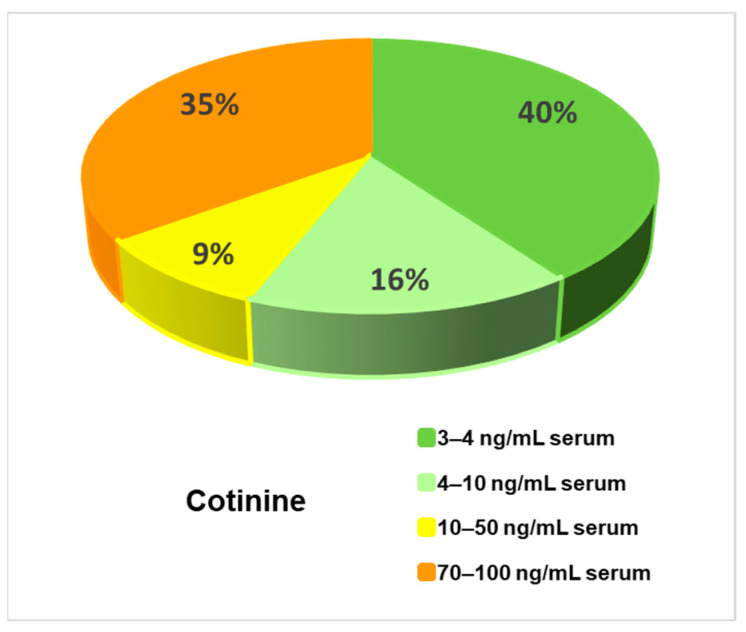
The distribution by serum cotinine levels of patients with mental disorders enrolled in the study.

The studied sample population, comprising 76 patients with mental disorders, was heterogeneous as regards the degree of exposure to tobacco smoke: 60% of patients were smokers and in 40% of patients low values of serum cotinine (<4 ng/mL serum) were measured, so they were exposed to a low amount of cigarette smoke and considered as non-smokers/passive smokers. However, an intermediate exposure to tobacco smoke, as reflected by serum cotinine levels ranging between 4 and 50 ng/mL serum, was found in 25% of smoker patients. The highest exposure to tobacco smoke was found in 35% of the subjects, who had serum cotinine values ranging between 70 and 100 ng/mL serum. This situation is depicted in Figure 1.

Therefore, a stratified analysis, according to tertile serum cotinine subgroups, was performed with the purpose of evaluating the status of oxidative stress biomarkers in relation to the degree of tobacco smoke exposure: lowest, medium, and highest cigarette smoke exposure. The patients were divided into three equal subgroups which displayed increasing mean values of serum cotinine (Table 2). Instead of dividing the patients into two unequal groups of smokers versus non-smokers, the approach through a stratified analysis using tertiles allowed us to more accurately examine the interrelationship be-tween the degree of patients’ exposure to cigarette smoke and oxidative stress biomarkers evaluated in the serum.

Mean values of serum cotinine levels were significantly increased among the tertile subgroups illustrating the lowest (tertile-1), the medium (tertile-2) and the highest (tertile-3) degree of exposure to cigarette smoke of the patients. Non-significant differences between tertiles were observed for the total antioxidant capacity of plasma measured as FRAP, whereas GSH levels were slightly, but also non-significantly, increased across tertiles. Conversely, AOPP levels were non-significantly lower in subjects from tertile-3 in comparison with the tertile-1 and tertile-2 subgroups (Table 2).

**Figure 2:**
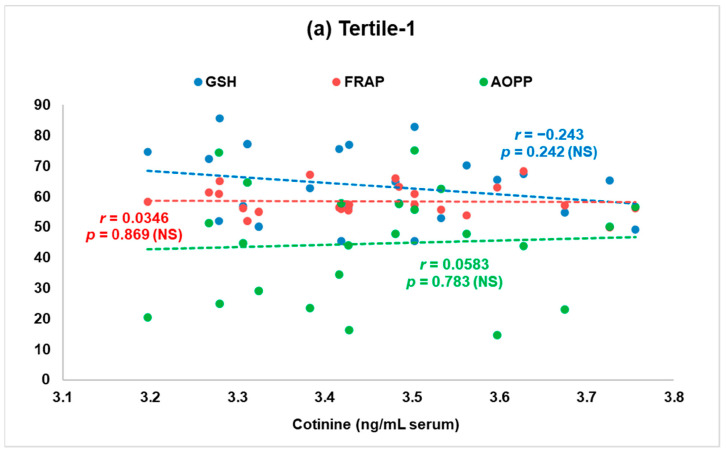

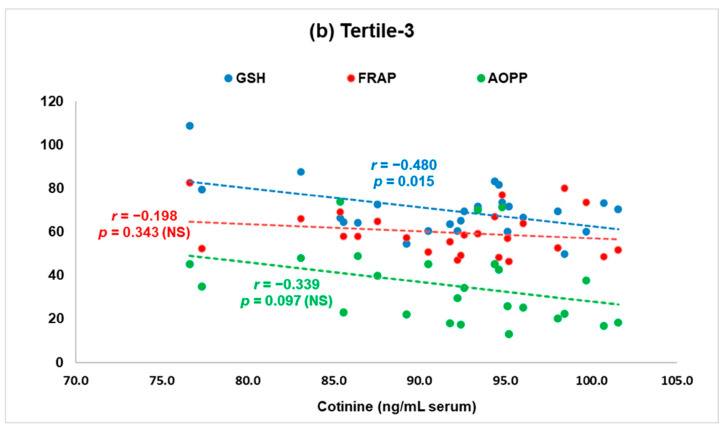
Pearson’s correlation (r) between serum glutatione (GSH), ferric reducing ability of plasma (FRAP), advanced oxidation protein products (AOPP), and smoking exposure measured as serum cotinine levels, in patients with serum cotinine levels <4 ng/mL serum (illustrating the lowest exposure to tobacco smoke, tertile-1, n = 25) (**a**) versus patients with cotinine levels >70 ng/mL serum (illustrating the highest exposure to tobacco smoke, tertile-3, n = 25) (**b**). NS: non-significant.

As the individual variability is very large in the second subgroup (tertile-2), with a large standard deviation of the mean cotinine values, we have made a further analysis of the interrelationships between smoking exposure -antioxidant defense-oxidative stress biomarkers, only for the subjects included in the tertile-1 and tertile-3 subgroup, which could characterize actually the passive smokers versus active smokers. The Pearson’s correlations (r) of serum cotinine with GSH, FRAP, and AOPPs in subjects from the tertile-1 and tertile-3 subgroups are illustrated graphically in Figure 2a,b.

Hence, in the tertile-1 subjects—with the lowest exposure to tobacco smoke—we observed a non-significant negative association between degree of nicotine exposure expressed as serum cotinine levels and GSH, as well as a positive correlation with oxidized proteins (AOPPs) (Figure 2a). In the tertile-3 subjects—with the highest exposure to tobacco smoke—we found a significant negative association between degree of nicotine inhalation, measured as serum cotinine levels and GSH. Furthermore, we found a non-significant negative association between tobacco exposure smoke and AOPPs (Figure 2b).

**Figure 3:**
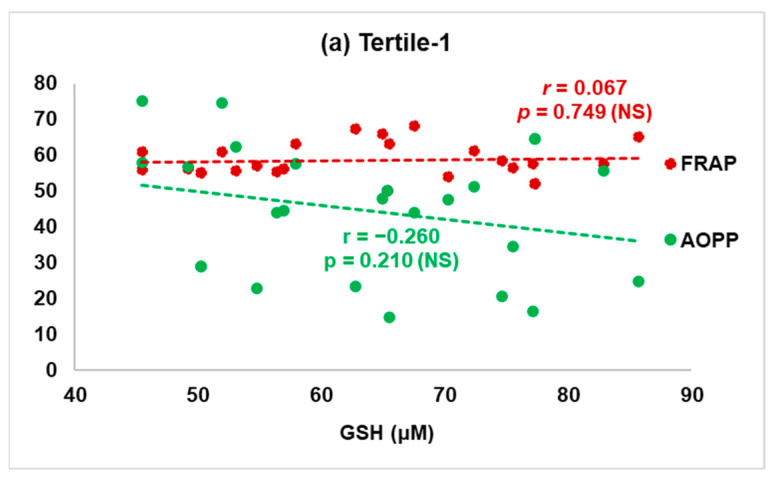

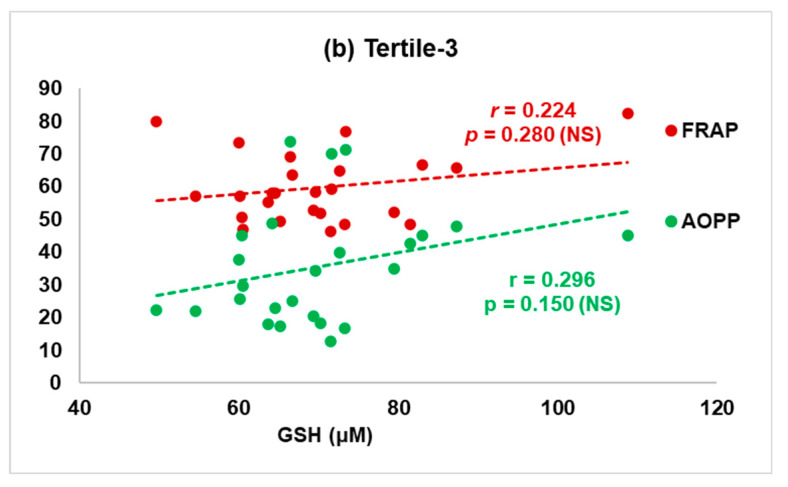
Pearson’s correlation (r) between ferric reducing ability of plasma (FRAP) and advanced oxidation protein products (AOPPs) with serum glutatione (GSH) levels in patients with the lowest exposure to tobacco smoke (tertile-1, n = 25) (**a**) versus patients with the highest exposure to tobacco smoke (tertile-3, n = 25) (**b**). NS: non-significant.

An additional correlation analysis was made between serum antioxidant defence and oxidative stress biomarkers in the tertile-1 and tertile-3 subgroups. The Pearson’s correlations (r) of FRAP and AOPPs with serum glutathione (GSH) in subjects from tertile-1 and tertile-3 are illustrated graphically in Figure 3a,b.

The correlation analysis showed that in the subgroup with the lowest tobacco smoke exposure (tertile-1 cotinine levels) the GSH values were inversely associated with the advanced protein oxidation biomarker (AOPPs), whereas in the subgroup of patients with the highest cotinine levels (tertile-3), we found a positive association between GSH levels and AOPPs, even though it was not significant. A slight positive non-significant association between GSH and total antioxidant status (FRAP) was also found in the tertile-3 subgroup.

## 4. Discussion

It is acknowledged that lifetime smoking rates are higher in patients who are diagnosed with major depression disorder (59%), bipolar disorder (83%), or schizophrenia and other mental disorders (90%), compared to 32% among adults with no mental illness [25]. Studies have indicated that exposure to cigarette smoke and/or nicotine, through active and passive smoking, causes a complex dysregulation of the sympathetic nervous system and immune system, as well as disruption of physiological oxidant/antioxidant homeostasis, all of which play a key role in the aggravation of a broad spectrum of diseases, including mental disorders [12,13,16,26].

There is a concern whether patients with mental disorders report their true smoking status, as mental health professionals, who only focus on mental disorders and addictions that have a direct impact on behaviors, disregard smoking addiction [13]. Therefore, tobacco is usually integrated in the health-care system functioning, especially in public hospitals. For this reason, in the present study the serum cotinine levels—a marker of nicotine exposure—were assessed for each subject, with the purpose of distinguishing active smokers from passive smokers/non-smokers, and to examine the interrelationships between cigarette smoke exposure and systemic antioxidant defence—oxidative stress biomarkers.

The studied sample population was heterogeneous regarding the individual values of serum cotinine, therefore, the patients were stratified in tertile subgroups. Comparison of the tertile subgroups showed significant differences in the degree of tobacco smoke exposure between subjects. The systemic antioxidant defence–oxidative stress biomarkers evaluated were: (1) reduced glutathione (GSH), a hydrosoluble antioxidant molecule with an important role in maintaining the cell and blood redox homeostasis; (2) the serum total antioxidant status, measured as the ferric reducing antioxidant power (FRAP); and (3) the advanced oxidation of the proteins (AOPPs), a marker which illustrates the degree of the serum protein damage as a result of myeloperoxidase (MPO) activity, the enzyme which is dynamically involved in the process of phagocytosis.

It is to be expected that if serum cotinine levels have increased—which is an indi-cator that nicotine exposure and smoke particulate inhalation has also increased—then the consequence will be a depletion in antioxidant defense molecules such as glutathione and reducing equivalents such as FRAP. On the other hand, markers of oxidative stress damage such as AOPPs, would be expected to increase along with increased particulate inhalation.

The most important finding of this study is the fact that the degree of tobacco smoke exposure, evaluated as serum cotinine levels, was inversely associated with GSH levels in both tertile-1 and tertile-3 (67% of subjects), in both passive and active smokers being statistically significantly lower in patients who had the highest exposure to tobacco smoke: tertile-3, active smokers. This negative correlation could suggest that nicotine toxicity is accompanied by a GSH depletion at a systemic level.

Our results are in line with old and recent studies: as a major endogenous antioxidant, GSH is thought to play a major role in the protection against oxidative stress in the lungs and other tissues [27,28,29,30]. Plasma and lung epithelial GSH concentrations have been shown to be decreased in association with smoking. This decrease is driven, in part, by conversion to its oxidized form (GSSG) [27]. On the other hand, smokers had higher concentrations of GSH in erythrocytes compared to non-smokers [27]. This situation is considered to be the result of an adaptive response of the body to continuous exposure to pro-oxidants present in tobacco smoke [27]. Recently, GSH levels in smokers who stopped smoking were reported to be significantly higher than in those who continued to smoke [28]. In non-smokers, and smokers who smoked less than 20 cigarettes per day, plasma GSH concentrations were comparable, in contrast to smokers of more than 20 cigarettes per day, who had significantly lower GSH levels [29]. Furthermore, the level of GSH is significantly lower in the saliva of subjects who consume forms of chewing tobacco compared to non-consumers [30].

Contrary to expectations, in the tertile-3 group, we paradoxically recorded the lowest AOPPs mean values, which were associated non-significantly with the degree of exposure to tobacco smoke, measured as serum cotinine. Our data showed that in the group with minimal exposure to tobacco smoke (tertile-1), the individual values of AOPPs decreased along with the increase in GSH levels, suggesting that at the systemic level the redox homeostasis is maintained and GSH exerts its antioxidant effect. By contrast, in smokers (tertile-3) this redox homeostasis is disturbed and GSH can no longer exert its antioxidant role. As we noticed, there is even a positive non-significant correlation between individual AOPPs values and serum GSH concentrations. We also noticed that the total antioxidant power of the serum determined as FRAP was not significantly changed across tertiles.

Some authors state the fact that tobacco and e-cigarette smoking exposure do not acutely alter the response of the antioxidant system—assessed as total antioxidant capacity (TAC), catalase activity (CAT), and reduced glutathione (GSH)—neither under active nor passive smoking conditions [31]. While these findings suggest potential anti-oxidative stress and anti-inflammatory effects, it is crucial to note that the lack of acute correlation between nicotine exposure and changes in the antioxidant system does not necessarily imply a protective effect. However, some studies claimed procognitive and neuroprotective effects of nicotine in neurodegenerative conditions such as Alzheimer’s disease, Parkinson’s disease, and schizophrenia [32,33,34,35,36,37].

Most of the patients included in our study had severe mental disorders, such as schizophrenia. Although GSH levels were lower in the first tertile (passive smokers) compared to the third tertile (active smokers), the differences did not reach statistical significance. This fact could be correlated also with the existence of a disturbance of the antioxidant–prooxidant balance determined by the mental disorders present in all patients. Reports in the literature show that major depressive disorder is accompanied by an increase in oxidative stress and a decrease in antioxidant capacity, leading to neuronal damage [38,39,40,41,42]. In a study conducted on 341 subjects, which combined major depressive disorder and nicotine dependence, it was shown that depressed smokers had significantly higher levels of nitric oxide metabolites (NOx), fibrinogen, high-sensitivity C-reactive protein (hs-CRP), and AOPPs, and lower levels of total reactive antioxidant potential (TRAP), compared to non-depressed patients who had never smoked [15].

These new results extend our knowledge on the association between systemic antioxidant status and oxidative stress markers in smoking subjects with mental disorders. Although the serum oxidative stress biomarkers GSH, FRAP, and AOPP do not cover all of the antioxidant molecules and oxidative damage processes existing at the systemic level, we provide new insights and information regarding the complex relationships within the triad tobacco smoking exposure–redox homeostasis–mental disorders.

The main limitation of the study is the small population sample studied. Moreover, taking into account the small number of subjects, any significantly sociodemographic and clinical differences between tertile subgroups were identified. Another limitation of the study is the reduced number of oxidative stress biomarkers evaluated.

## 5. Conclusions

The present study identified in subjects with mental disorders significant associations between the degree of tobacco smoke exposure evaluated as serum cotinine levels, and total antioxidant status/oxidative stress biomarkers. The exposure to smoke particulate components was accompanied by a GSH depletion at systemic level and a disturbed redox homeostasis but also exerted an inhibitory effect on protein oxidation via myeloperoxidase action. However, these biomarkers cannot be used as the exclusive indicators of systemic oxidative stress, with further studies being necessary to understand the unknown factors affecting the global antioxidant status during chronic cigarette smoke exposure. To gain physicians’ interest in new techniques of oxidative stress assessment is crucial for the understanding of nicotine’s and smoke particulate components’ involvement in the evolution of diseases in which oxidative stress appears to play an important role. This would increase the quality of life of patients with mental disorders addicted to tobacco consumption.

## Figures and Tables

**Table 1:** Socio-demographic and clinical characterization of the study group.

Parameter	Group Characteristics
Total number of patients	76
Distribution by sex	61 males (80.26%), 15 females (19.74%)
Male/female ratio	4.06
Average age (±SD)	50.00 ± 8.8 years (range 25–40)
Male average age (±SD)	45.43 ± 9.77 years (range 25–62)
Female average age (±SD)	50.6 ± 10.48 years (range 39–77)
Diagnosis	Paranoid schizophrenia (46.05%, 35/76 cases); mixed background organic personality disorders (21.05%, 16/76 cases); acute mental disorder (22.37%, 17/76 cases); others (10.53%, 8/76 cases)
Treatment	Carbamazepine; amisulpride; haloperidol; levomepromazine; aripiprazol; memantine; clozapine; tiapride; quetiapine; diazepam; bromazepam

**Table 2:** Oxidative stress parameters in patients with mental disorders (n = 76) across tertile subgroups of serum cotinine level.

Variable	Tertile-1 (n = 25)	Tertile-2 (n = 26)	Statistical Significance vs. Tertile-1 Group		Tertile-3 (n = 25)	Statistical Significance vs. Tertile-1 Group		Statistical Significance vs. Tertile-2 Group	
	Mean ± SD	Mean ± SD	*p*-Value	*t*-Value	Mean ± SD	*p*-Value	*t*-Value	*p*-Value	*t*-Value
Cotinine (ng/mL serum)	3.44 ± 0.15	15.86 ± 20.55	0.004 *	−3.022	91.71 ± 6.57	0.000 **	67.158	0.000 **	17.574
Oxidative stress parameters									
GSH, µM	63.62 ± 12.07	66.93 ± 10.11	0.297	−1.053	69.91 ± 11.93	0.345	0.953	0.069	1.854
FRAP, mM	58.44 ± 4.92	58.91 ± 11.25	0.838	−0.191	59.73 ± 10.43	0.579	−0.559	0.790	−0.267
AOPPs, µM	44.58 ± 18.07	41.84 ± 13.56	0.544	0.606	35.52 ± 17.53	0.078	−1.799	0.160	−1.426

GSH, glutathione; FRAP, ferric reducing ability of plasma; AOPPs, advanced oxidation protein products; SD, standard deviation; n, number of subjects. Statistical significance: * *p* < 0.01; ** *p* < 0.001.

## Data Availability

Data are contained within the article.
